# Rapid Proficiency in Femoral and Popliteal Nerve Identification Is Achievable After a Brief Educational Intervention

**DOI:** 10.7759/cureus.76807

**Published:** 2025-01-02

**Authors:** Ashley Grant, Colin Sullivan, Brandon Buchel, Charlotte Derr

**Affiliations:** 1 Emergency Medicine, Florida State University College of Medicine, Sarasota, USA; 2 Emergency Medicine, St Joseph's Hospital - South, Riverview, USA; 3 Emergency Medicine, University of South Florida Morsani College of Medicine, Tampa, USA

**Keywords:** educational intervention, femoral, identification, nerves, popliteal, proficiency, regional anesthesia, sonography, ultrasound

## Abstract

Introduction

Ultrasound guidance for the delivery of regional anesthesia in the emergency department (ED) is an area of increasing interest. However, in order to perform a regional nerve block, one must first be able to correctly identify the specific nerve on ultrasound. Therefore, the primary purpose of this study is to investigate the number of supervised ultrasound examinations required for an emergency medicine (ED) physician to gain proficiency in accurately identifying the nerves of the lower extremity at the level of the femoral and popliteal nerves.

Methods

The proficiency outcome was defined as the number of attempts a resident needed to locate and correctly identify the femoral and popliteal nerves for 10 consecutive examinations. Didactic education was provided via a one-hour lecture and two supervised hands-on ultrasound examinations for each region prior to testing. Count data are summarized using percentages or medians and ranges. Random effects negative binomial regression was used for modeling panel count data, with the results summarized as an incidence rate ratio (95% confidence interval) and P-values <0.05 considered statistically significant.

Results

Complete data for the number of attempts, confidence, gender, and years in practice was available for 31 residents. The median number of attempts and range for popliteal and femoral nerves were 0 (0-11) and 0 (0-11), respectively. The median level of confidence and range for popliteal and femoral nerves were 3.5 (1-5) and 3 (1-5), respectively. There was a significant association between confidence and proficiency (P=0.009) and between years in practice and proficiency (P=0.010).

Conclusion

The findings of this small data set would suggest that proficiency can be quickly obtained in identifying the femoral and popliteal nerves with ultrasound after only a few proctored examinations. A significant association was found between years in practice and proficiency. This suggests that physicians with previous ultrasound scanning experience may require fewer supervised examinations when learning how to identify the femoral and popliteal nerves.

## Introduction

Providing adequate pain control has become an area of increasing focus for emergency physicians. It is not only an important aspect of the compassionate care we provide for our patients in the emergency department (ED) but it is also considered a core measure and tied to reimbursement [1]. Not surprisingly there is a rising interest in the role of ultrasound as a tool for the delivery of analgesia by emergency medicine (EM) providers [2,3]. With point-of-care ultrasound strongly supported by an American College of Emergency Physicians (ACEP) clinical policy, the notion of ultrasound-guided regional anesthesia by the EM provider is a logical next step [4-7]. Ultrasound-guided regional anesthesia is already a very well-established modality of pain control in the anesthesia community where it has been proven to be safe, effective, and efficient with relatively few contraindications or complications [8-11]. Recently this modality has also been explored in the acute traumatic setting in the treatment of upper and lower extremity injuries including hip and shoulder injuries, in addition to rib fractures [12-16].

At this time, opioids remain the most common means of acute pain control and will no doubt remain a valuable adjunct in the treatment of acute injuries. Regional anesthesia has the ability to decrease the use of these medications, with many resultant benefits to both patients and providers, including improved pain control and decreased adverse effects such as delirium, sedation, hemodynamic effects, constipation, and nausea [17,18]. Taken further, this can also potentially be extrapolated to decreased length of hospital stay and decreased risk of opioid dependence and would be of particular interest in special populations such as pediatrics and geriatrics [13,17-23].

Historically, regional nerve blocks have been performed using landmark-based techniques with or without the concomitant use of nerve stimulators. In current literature, there is strong support for the use of ultrasound guidance alone or in combination with the use of nerve stimulators [8-11]. These techniques have been reported in EM literature for lower extremity applications within various healthcare systems around the world [22-26]. It has further been proposed that ultrasound-guided anesthesia could potentially be of utility in disaster and resource-poor settings [27].

Proficiency in regional anesthesia techniques for the femoral and popliteal nerves enables emergency room physicians to effectively manage pain, perform necessary procedures, and improve patient outcomes in a variety of emergency situations involving lower extremity injuries and trauma. At the core of ultrasound-guided peripheral nerve blocks is knowledge of the underlying neuroanatomy and the ability to reliably sonographically identify the appropriate structure. Therefore, the primary objective of this study is to determine how many supervised peripheral nerve examinations are necessary for an EM physician to become proficient and confident in accurately locating and identifying the correct neuroanatomy of the lower extremity, specifically the femoral and popliteal (posterior tibial and peroneal) nerves. The number of such studies that would confer proficiency in correctly identifying the nerves of the lower extremity has yet to be established, though there has been some research aimed at teaching the overall fundamentals of the ultrasound-guided femoral nerve block [28-30]. Therefore, this study focuses on providing more objective evidence regarding the number of supervised peripheral nerve examinations that are necessary to proficiently accomplish the crucial step of sonographically identifying the appropriate neuroanatomy.

## Materials and methods

This study was constructed as a prospective, interrupted, time-series design without a control group and was formally approved by the Institutional Review Board prior to its implementation. The participants in the study were current residents enrolled in the ED residency program at the University of South Florida Morsani College of Medicine in Tampa, Florida. The study took place over the course of a year. The resident participation in this study was voluntary and informed consent was obtained prior to the start of the study. Due to the descriptive nature of this study, formal sample size calculations were not performed. The residency program enrolls 10 residents per year and is a three-year program.

Residents were given an introductory femoral and popliteal nerve block didactic lecture (approximately one hour in length) followed by two supervised hands-on ultrasound examinations for how to identify each nerve. Next, an initial objective structured clinical examination (OSCE) was performed to evaluate their ultrasound skills and ability to identify the femoral and popliteal nerves. Over the next seven months, the residents were periodically re-evaluated via OSCE. Participants did not undertake an additional review of ultrasound images from textbooks or online resources between the initial didactic session, supervised examinations, and subsequent assessments. Prior to performing each OSCE session, participants completed a self-assessment tool (Table 1) regarding their confidence in correctly locating and identifying peripheral nerve anatomy with the use of ultrasound. Next, their ability to correctly locate and identify the peripheral nerves using ultrasound was confirmed by an emergency physician sonologist experienced in the visualization of peripheral nerves of the lower extremity.

**Table 1:** Peripheral Nerve Study Self-Assessment Survey For each exam above, please indicate your level of confidence in peripheral nerve identification, specific to the area being assessed, using ultrasound. 1- I always have difficulty 2 - I often have difficulty 3 - I sometimes have difficulty 4 - I rarely have difficulty 5 - I never have difficulty

	Exam # Date	01	02	03	04	05	06	07	08	09	10	11	12	13	14	15	16	17	18	19	20
Exam Type																					
Popliteal Nerves																					
Femoral Nerve																					

The residents’ performance was recorded using a data collection tool (Table 2). The data collected was then analyzed to determine the median number of exams required for an EM physician to become proficient and feel confident in his or her ability to correctly identify the femoral and popliteal nerves. Study participants were excluded if they were determined to have individually completed more than 10 peripheral nerve examinations prior to the start of the study or if they simply did not wish to participate in the study. No participants met the exclusion criteria. 

**Table 2:** Peripheral Nerve Study Data Collection Tool

Study ID: PGY:	Exam Number/Nerve Correctly Identified?																			
Nerve to Identify	1	2	3	4	5	6	7	8	9	10	11	12	13	14	15	16	17	18	19	20
Femoral Nerve	Y N	Y N	Y N	Y N	Y N	Y N	Y N	Y N	Y N	Y N	Y N	Y N	Y N	Y N	Y N	Y N	Y N	Y N	Y N	Y N
Popliteal Nerve	Y N	Y N	Y N	Y N	Y N	Y N	Y N	Y N	Y N	Y N	Y N	Y N	Y N	Y N	Y N	Y N	Y N	Y N	Y N	Y N

The primary study endpoint was the ability to correctly locate and identify, 100% of the time, the femoral and popliteal nerves, as listed in the data tool (Table 2). The resident needed to demonstrate this for 10 consecutive peripheral nerve examinations to be considered proficient. The secondary study endpoint is the measurement of the resident’s confidence (Table 1) in their ability to correctly locate and identify, 100% of the time, all of the peripheral nerve anatomy listed in the data tool.

A negative binomial regression coefficient was used to describe the data. We followed the STROBE guidelines for reporting observational studies. The models for the OSCE examinations were volunteer members comprised of patients within the ED, medical students, family members, and nurses. Sonosite ultrasound machines (SonoSite Inc - US, Bothell, Washington 98021) were used in the study. The imaging was performed using two high-frequency linear array transducers: the Sonosite L25x (6-13MHz) and the Sonosite HLF38xi (6-13 MHz). 

Descriptive statistics are reported as median (range) for continuous variables and as frequencies and percentages for categorical variables. Random effects negative binomial regression was used for modeling panel count data. Negative coefficients indicated an increase in proficiency. The results are summarized as the incidence rate ratio (95% confidence interval). P-values <0.05 were considered statistically significant. The analysis was performed using Stata 13.1.

## Results

The proficiency outcome was defined as the number of attempts required for a resident to correctly locate and identify 100% of the peripheral nerve anatomy on 10 consecutive examinations. Count data are summarized using percentages or medians and ranges. Random effects negative binomial regression was used for modeling panel count data. P-values <0.05 were considered statistically significant.

Complete data for the number of attempts, gender, and PGY training was available for 31 residents. Sixteen male and 15 female residents performed examinations. The median years in practice was two (range: one to three), with 11 (35%) in year 1, 11 (35%) in year 2, and nine (30%) in year 3. The median number of attempts and range for popliteal and femoral nerves were 0 (0-11) and 0 (0-11), respectively. The self-reported confidence ratings of each individual resident were marked prior to the completion of each OSCE examination for each nerve separately. The value range of the confidence scale was set from 1 (not at all confident) to 5 (very confident). The median level of confidence and range for popliteal and femoral nerves were 3.5 (1-5) and 3 (1-5), respectively. This data was recorded and analyzed (Table 3) with negative binomial regression for the outcome proficiency being performed to identify significantly associated variables. Negative coefficients indicate an increase in proficiency. Negative binomial regression identified a significant association between confidence and proficiency (P=0.009) and between years in practice and proficiency (P=0.010).

**Table 3:** Incidence Rate Ratios Based on Negative Binomial Regression Coefficients for Gender, Years in Practice, Confidence, and Nerve Type for 31 Residents

	Incidence Rate Ratio (95% Confidence Interval)	P-value
Gender (vs. Female)	1.94 (0.64-5.86)	0.24
Years in Practice (vs. 1 and 2)	0.65 (0.19-2.23)	0.49
Years in Practice (vs. 3)	0.07 (0.08-0.66)	0.020
Confidence	1.34 (0.80-2.22)	0.27
Nerve (vs. Popliteal)	0.47 (0.18-1.18)	0.11

## Discussion

Proficiency in the identification of the peripheral nerves within the leg, specifically the femoral and popliteal nerves, was found to be attainable within a reasonably small number of proctored OSCEs following one hour of formalized training and two assisted ultrasound examinations. No further education was necessary to locate and successfully identify the femoral and popliteal nerves for 10 consecutive examinations for the majority of residents involved in this study. The maximum number of attempts at identifying the popliteal and femoral nerves 10 consecutive times by any participant was 21. The maximum number of assisted OSCE attempts to reach proficiency, per our definition of 100% accuracy in 10 consecutive examinations, for the popliteal and femoral nerves was 11, meaning that it took 11 examinations before the resident then went on to identify the popliteal nerve successfully in the next 10 examinations. This was the same for the femoral nerve. Eleven residents were unable to consecutively identify 10 popliteal nerves correctly from their first attempt, whereas only seven residents were unable to consecutively identify the femoral nerve 10 times from their first attempt. The study also found that the more years of training a resident had the more proficient they were in identifying the femoral and popliteal nerves correctly and consecutively.

As point-of-care ultrasound has become a standard in EM training, the various uses of this technology as well as the situations in which it may be utilized has expanded. This is especially true in regard to its use in procedures where ultrasound not only can increase the efficiency of the task at hand but also decrease complications. Not surprisingly, previous studies regarding formalized education and proficiency in using bedside ultrasonography in the realm of regional anesthesia were most often found within the anesthesia literature [29].

However, EM has more recently begun to do its part in contributing to this data. That being said, there has yet to be a formalized study that specifically concentrates on defining proficiency in the identification of the anatomy involved in these procedures. This is what our study set out to accomplish.

Currently, the ACEP does not set a recommended standard of competency specific to regional anesthesia. As this procedure becomes more of an expected skillset in EM, it will become increasingly important to define the level at which a physician is deemed competent and capable of safely performing regional anesthesia. We believe that before a reasonable number of completed procedures to demonstrate competency is devised, formal education and evaluation of all aspects of these procedures, including simply locating the anatomical structures involved, is important. Hands-on experience with real patients with direct supervision is also essential, as it not only facilitates skill acquisition but also builds confidence and competence in performing these procedures.

A small sample size is one limitation identified in this study. This resulted in only descriptive analysis of the data being possible due to limited power in the number of participants that we had available to pull from. Being that the data was collected within a single academic year we were limited to 31 residents to participate in the study. That being said, the size of the EM residency at the University of South Florida Morsani College of Medicine is similar to many other residency programs across the country. It should be noted that at the time of the study, an additional resident was present secondary to an interruption in training time for medical leave. A second limitation is the potential for differences in technical skills due to varying levels of training. We acknowledge that residents at different PGY levels may have differing baseline technical skills due to the cumulative experience and training acquired over the course of their residency. Future studies could stratify participants by PGY level or include a pre-intervention skill assessment to better account for baseline differences. This would help further delineate the specific impact of supervised ultrasound training on skill acquisition.

## Conclusions

Minimal supervised training is required before EM physicians can reliably identify the femoral and popliteal nerves by ultrasound. There is an association between years in practice and proficiency suggesting that this is reached more readily in individuals who already have basic ultrasound experience. Therefore, peripheral nerve identification and the task of providing regional anesthesia might be considered a more advanced application that is taught after an initial introductory ultrasound course and the development of basic ultrasound skills. It has been our experience that by using a structured training curriculum of didactic lectures and supervised hands-on practice, residents can become proficient and confident at identifying the femoral and popliteal nerves with the use of ultrasound after only a small amount of formalized instruction.

Regional anesthesia is a skill set within the realm of EM. The correct identification of neuronal structures by ultrasound is just the first step in teaching regional anesthesia. Before becoming proficient at performing regional nerve blocks, additional education and supervision are required, specifically in the areas of equipment selection, needle insertion techniques, the injection of regional anesthetic solutions and their pharmacokinetics, and patient safety. Supervised practice on real patients is essential for physicians to develop proficiency in ultrasound-guided regional anesthesia. Identifying nerves in real time may present greater challenges compared to an OSCE setting, as it requires navigating anatomical variations and mastering the technical complexities of needle guidance. A future study will aim to expand upon this study and serve to assess the physician’s ability (number of procedures completed to define competency) to adequately perform lower extremity regional anesthesia following this additional education.
